# The Effects of Low Levels of Dystrophin on Mouse Muscle Function and Pathology

**DOI:** 10.1371/journal.pone.0031937

**Published:** 2012-02-16

**Authors:** Maaike van Putten, Margriet Hulsker, Vishna Devi Nadarajah, Sandra H. van Heiningen, Ella van Huizen, Maarten van Iterson, Peter Admiraal, Tobias Messemaker, Johan T. den Dunnen, Peter A. C. 't Hoen, Annemieke Aartsma-Rus

**Affiliations:** Department of Human Genetics, Leiden University Medical Center, Leiden, the Netherlands; Johns Hopkins Univ. School of Medicine, United States of America

## Abstract

Duchenne muscular dystrophy (DMD) is a severe progressive muscular disorder caused by reading frame disrupting mutations in the *DMD* gene, preventing the synthesis of functional dystrophin. As dystrophin provides muscle fiber stability during contractions, dystrophin negative fibers are prone to exercise-induced damage. Upon exhaustion of the regenerative capacity, fibers will be replaced by fibrotic and fat tissue resulting in a progressive loss of function eventually leading to death in the early thirties. With several promising approaches for the treatment of DMD aiming at dystrophin restoration in clinical trials, there is an increasing need to determine more precisely which dystrophin levels are sufficient to restore muscle fiber integrity, protect against muscle damage and improve muscle function.

To address this we generated a new mouse model (*mdx-Xist* ^Δhs^) with varying, low dystrophin levels (3–47%, mean 22.7%, stdev 12.1, *n* = 24) due to skewed X-inactivation. Longitudinal sections revealed that within individual fibers, some nuclei did and some did not express dystrophin, resulting in a random, mosaic pattern of dystrophin expression within fibers.

*Mdx-Xist* ^Δhs^, *mdx* and wild type females underwent a 12 week functional test regime consisting of different tests to assess muscle function at base line, or after chronic treadmill running exercise. Overall, *mdx-Xist* ^Δhs^ mice with 3–14% dystrophin outperformed *mdx* mice in the functional tests. Improved histopathology was observed in mice with 15–29% dystrophin and these levels also resulted in normalized expression of pro-inflammatory biomarker genes, while for other parameters >30% of dystrophin was needed. Chronic exercise clearly worsened pathology, which needed dystrophin levels >20% for protection. Based on these findings, we conclude that while even dystrophin levels below 15% can improve pathology and performance, levels of >20% are needed to fully protect muscle fibers from exercise-induced damage.

## Introduction

Duchenne Muscular Dystrophy (DMD) is an X-linked recessive disorder affecting 1∶3500 new born boys. It is characterized by muscle fiber wasting, functional impairment and eventual death due to respiratory and heart failure. The underlying causes are frame-shifting and nonsense mutations in the *DMD* gene, resulting in the absence of functional dystrophin protein. Intact dystrophin anchors the intracellular cytoskeleton to the extracellular matrix and thereby prevents membrane damage during muscle contraction [1], [2]. An allelic, less severe form of the disease, Becker muscular dystrophy (BMD) is caused by mutations that maintain the open reading frame and allow synthesis of internally deleted, partially functional dystrophin proteins [3], [4].

There is no cure for DMD, but many potential therapeutic compounds currently tested in clinical trials aim at restoration of (a BMD like) dystrophin [5]–[12]. These trials at best resulted in the synthesis of low levels of dystrophin protein. However, it is not yet known how these levels will affect disease pathology, and which levels are needed to maintain muscle fiber integrity, to prevent against exercise-induced damage, or to improve muscle function. In addition, it is as yet unknown whether low dystrophin levels will stabilize and/or delay disease progression [8]. In perspective of further optimization of currently tested potential therapeutic compounds, detailed studies in this direction are necessary.

Early studies primarily involved female *DMD* carriers, heterozygous *mdx* mice and isolated BMD patients expressing less than 50% dystrophin. Female *DMD* mutation carriers express dystrophin in approximately 50% of the fibers (when seen in a transverse cross section) due to random X-inactivation early in life [13], [14]. During life, the proportion of dystrophin positive fibers increases due to positive selection. It appears that this is sufficient to maintain skeletal muscle function and fiber integrity in both human and mice. Although skeletal muscles appear to escape damage, human carriers are at risk for *DMD*-associated dilated cardiomyopathy [15], while no heart failure is observed in heterozygous *mdx* mice [16], [17]. Nevertheless, symptomatic carriers have been described, often expressing less than 50% of normal dystrophin levels due to either gross chromosomal rearrangements [18], or unfortunate skewed X-inactivation in which the degree of skewedness correlated with disease severity [13], [19]–[21]. In addition, BMD patients generally have lower levels of dystrophin and these levels seem to correlate with disease severity, where levels <10% are observed in very severe patients and levels >20% in moderate/mild patients [4], [22]. Based on a case study involving one patient, it appears that dystrophin levels as low as 30% can be sufficient to largely prevent a muscle phenotype [23]. The amount of revertant fibers has also been reported to correlate positively with disease severity [24]. However, reports on BMD and skewed X-inactivation cases involve low numbers of patients and detailed analysis (e.g. assessment of dystrophin levels in various muscles) is not possible for obvious reasons. Thus, there is an increasing need for a mouse model expressing low levels of dystrophin to allow the detailed study of the effects of low dystrophin levels on disease pathology.

So far, several attempts have been made to achieve this. Unfortunately, each had limitations. First, transgenic mice expressing low levels of full-length or truncated dystrophin of murine or human origin have been generated to test whether this results in a less severe phenotype [25], [26]. However, from the 13 mouse lines generated, only two expressed dystrophin levels lower than 50% of wild type in both the quadriceps and diaphragm. The other lines had higher dystrophin levels or a combination of both higher and lower levels than wild type. From the two potential lines, one expressed low dystrophin levels (15% quadriceps and <5% diaphragm), while the other line expressed higher dystrophin levels (40% quadriceps and 20% diaphragm), thus allowing detailed analysis of only two different dystrophin levels. Dunant et al. generated *mdx* mice in which dystrophin expression was driven by a 1.35-kb MCK promoter resulting in abundant expression in fast-twitch muscles (∼50%) and very low expression in slow-twitch fibers. The heart did not express dystrophin at all [27]. The fiber type specific dystrophin expression makes this model less suitable for detailed analysis. In another study, Stillwell et al. generated chimeric mice expressing various dystrophin levels depending on the amount of wild type cells incorporated in *mdx* blastocytes. Mice expressed dystrophin in skeletal muscles in a very high (60–100%) or low (<5%) manner [28]. This, combined with the fact that this method is extremely labour intensive, makes this approach less favourable for extended studies. The final model known to express low dystrophin levels is the *mdx^3cv^* mouse. Studies in the *mdx^3cv^* mouse revealed that even ∼5% of dystrophin increased specific tetanic force and partly prevented against eccentric contractions induced damage in young but not in old mice. However, it was insufficient to prevent histopathology, since severity did not differ from dystrophin negative *mdx^4cv^* mice [29]. Although *mdx^3cv^* mice are easy to generate, dystrophin levels do not exceed 5%, making studies on the effect of higher dystrophin levels e.g. 20–30% impossible.

**Figure 1:**
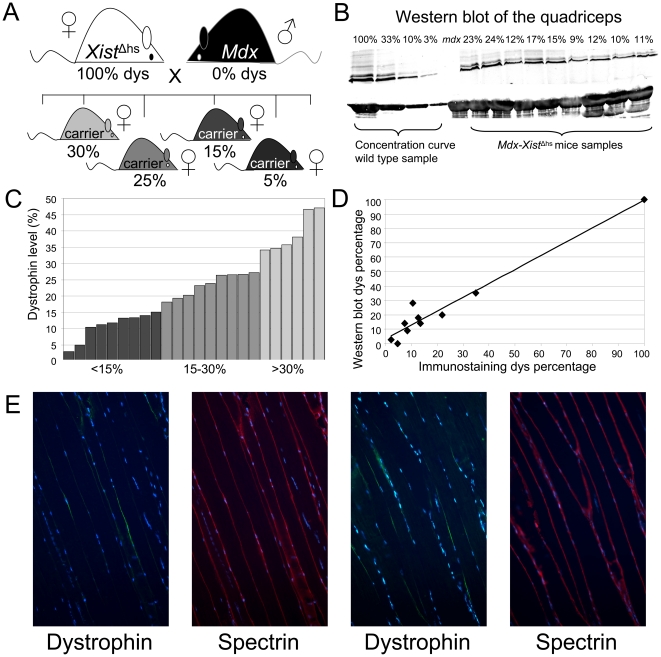
*Mdx-Xist* ^Δhs^ mice. A. To breed mice with low dystrophin levels, female *Xist* ^Δhs^ mice, carrying a mutation in the *Xist* promoter which coordinates X-inactivation, were crossed with dystrophin negative *mdx* males. During embryogenesis, the maternal X-chromosome encoding a functional dystrophin gene will be preferentially (60–90%) inactivated as a result of the mutated *Xist* promoter. The *Xist* ^Δhs^ mice were a kind gift from N. Brockdorff (MRC Clinical Sciences Center, London, UK, current affiliation Department of Biochemistry, University of Oxford, UK). B. Picture of a representative Western blot. The percentage of dystrophin was determined for the quadriceps of all *mdx-Xist* ^Δhs^ mice by Western blot (2–9 technical replicates per mouse). The percentage of individual mice was determined using a concentration curve made from wild type samples. Myosin was used as a loading control. C. Skewed X-inactivation resulted in dystrophin levels of 3–47% (mean 22.7, stdev 12.1, *n* = 24) (as determined by Western blot) in the female *mdx-Xist* ^Δhs^ offspring. Each bar represents the dystrophin level of an individual mouse. The dystrophin levels of the individual mice belonging to the three dystrophin groups can be appreciated from this graph. D. Dystrophin levels determined by Western blot and manual counting of dystrophin positive fibers demonstrate a strong correlation (R = 0.97). E. Longitudinal sections of a quadriceps stained with dystrophin (green) and spectrin (red). From the pictures it can be appreciated that dystrophin expression is not homogeneously expressed across the fiber but rather confined to certain nuclear domains.

Taken together, these models give an indication that <50% dystrophin is sufficient for improvement of many pathological aspects, however, they are not ideal to assess the effects of low dystrophin levels on disease pathology in detail. Our study describes a mouse model expressing low dystrophin levels, based on non-random X-inactivation. In mammals, X-inactivation is regulated by the X-inactivation center in which *Xist* is the key player [30], [31]. Mutations in the *Xist* promoter can skew the randomness of X-inactivation resulting in preferential inactivation (60–90%) of the X-chromosome containing the mutated *Xist* gene [32]. Here, non-dystrophic female *Xist* ^Δhs^ mice with a mutation in the *Xist* promoter were crossed with dystrophic *mdx* males. This resulted in female *mdx-Xist* ^Δhs^ mice in which the X-chromosome containing the wild type dystrophin is preferentially inactivated, leading to a wide range of low dystrophin levels in individual mice (Figure 1). Within individual muscle fibers of these pups, a mosaic pattern of dystrophin expression was observed, in which nuclei either did or did not express dystrophin. Experiments revealed that low dystrophin levels have a beneficial effect on muscle integrity, histology, function and serum biomarkers in a dose dependent manner.

## Results

### Mdx-Xist^Δhs^ mice express varying levels of dystrophin

To study whether low dystrophin levels reduce the dystrophic pathology of *mdx* mice, *mdx-Xist* ^Δhs^ mice carrying a wide range of low dystrophin levels based on non-random X-inactivation were bred (Figure 1A). For each *mdx-Xist* ^Δhs^ mouse, dystrophin levels in the quadriceps muscle were quantified by Western blot (Figure 1B). These varied between 3–47% of wild type levels (mean 22.7, stdev 12.1, *n* = 24) (Figure 1C), which correlated well (R = 0.97) with the percentages of dystrophin positive fibers in cross sections of the same muscle, as assessed by blinded manual counting (Figure 1D). Dystrophin levels of individual muscles differed (assessed in *n* = 6 *mdx-Xist* ^Δhs^ mice), with the tibialis anterior and biceps expressing slightly higher levels than the quadriceps, gastrocnemius and triceps. Lowest dystrophin levels were found in the diaphragm and heart (Figure S1A–B). The dystrophin level of the quadriceps was used to group the mice. Longitudinal sections of the quadriceps revealed that dystrophin expression along a single whole fiber was patchy, with nuclei either expressing dystrophin or not (Figure 1E). This indicates that dystrophin proteins cannot freely diffuse along the muscle fiber and that nuclei where the normal *DMD* gene is not inactivated serve only certain regional domains in the muscle fiber. Transverse cross sections of several skeletal muscles and heart revealed that dystrophin positive fibers were most often grouped together and that these small groups were spread over the muscle (Figure S1C).

Dystrophin is part of a larger complex of dystrophin-glycoprotein associated proteins, such as β-dystroglycan and nNOS. As expected, dystrophin positive fibers of the *mdx-Xist* ^Δhs^ mice were also positive for β-dystroglycan and nNOS (Figure S2A). In absence of dystrophin, the homolog utrophin can partly take over its function. Muscles of adult *mdx* mice have significantly higher utrophin levels than those of adult C57BL/10ScSnJ, as utrophin is overexpressed in the absence of dystrophin [33]. We assessed how dystrophin expression influences utrophin expression for six *mdx-Xist* ^Δhs^ mice expressing various levels of dystrophin. In *mdx-Xist* ^Δhs^ mice with very low dystrophin levels (<15%), utrophin levels were increased as confirmed by Western blot (Figure S2B, lane 2 and 6). In contrast, *mdx-Xist* ^Δhs^ mice with intermediate dystrophin levels (>15%) had low utrophin levels (lanes 1, 3–5). We did not observe a decrease in utrophin intensity in dystrophin positive fibers analyzed by immunohistology (data not shown).

### Dystrophin level dependent improvement in muscle function

**Figure 2:**
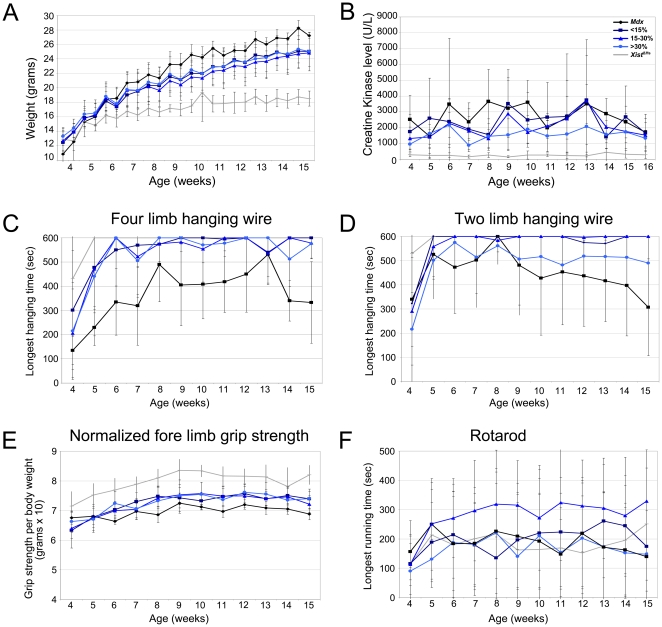
Body weight, CK levels and functional performance of the mouse models. A. Normalization towards wild type levels was observed for bodyweight of the *mdx-Xist* ^Δhs^ mice. At the end of the functional test regime *mdx* mice were significantly heavier (*P*<0.001) than the other mice. B. CK levels of *mdx* and *mdx-Xist* ^Δhs^ mice with low dystrophin levels were both significantly (*P*<0.001) higher compared to the levels of *mdx-Xist* ^Δhs^ mice with >30% dystrophin and *Xist* ^Δhs^ mice. C. Four limb hanging wire performance was comparable for the *Xist* ^Δhs^ and *mdx-Xist* ^Δhs^ mice, while *mdx* mice performed significantly (*P*<0.001) worse. D. The best performance on the two limb hanging wire test was obtained for the *Xist* ^Δhs^, *mdx-Xist* ^Δhs^ with <15% and 15–30% while the *mdx* and *mdx-Xist* ^Δhs^ with >30% dystrophin performed significantly (*P*<0.001) worse. Bad performance of *mdx-Xist* ^Δhs^ with >30% was due to the bad performance of one individual mouse. E. Normalized fore limb grip strength was significantly (*P*<0.001) higher in the *Xist* ^Δhs^ mice compared to the other strains and all the *mdx-Xist* ^Δhs^ mice were significantly (*P*<0.001) stronger than the *mdx* mice. F. No difference in rotarod running was observed, except for the *mdx-Xist* ^Δhs^ mice with 15–30% dystrophin, which performed significantly (*P*<0.001) better than all other groups.

To determine the effect of low dystrophin levels on functional performance, *Xist* ^Δhs^, *mdx* and *mdx-Xist* ^Δhs^ mice were subjected to a functional test regime consisting of four functional tests per week for 12 weeks, during which also body weight and creatine kinase (CK) levels were monitored. After sacrificing, quadriceps muscles were isolated and dystrophin levels were determined by Western blot. K-means clustering was used to divide *mdx-Xist* ^Δhs^ mice into three groups in an unbiased way. This resulted in the following division: <15%, 15–30% and >30% dystrophin. *Mdx* mice were significantly (*P*<0.001) heavier at the end of the functional test regime than the other mouse strains (mean weight 26, 24.5 and 18.2 gram for *mdx*, *mdx*-*Xist* ^Δhs^ and *Xist* ^Δhs^, respectively). An increased weight compared to wild type has been described before for *mdx* mice [34]. *Xist* ^Δhs^ mice had a stable body weight from week 11 onwards, while the other models showed a mild increase over time. The body weights of the *mdx-Xist* ^Δhs^ mice from the three dystrophin level groups were very similar and fell in between *mdx* and *Xist* ^Δhs^ mice, but significantly (*P*<0.001) differed from both (Figure 2A).

CK levels are a measure for muscle condition and are <500 U/L in healthy mice. CK levels of the *Xist* ^Δhs^ mice never exceeded this threshold and were, averaging 270 U/L, significantly (*P*<0.001) lower than levels of the other mouse strains. *Mdx* mice had the highest CK levels (average 2700 U/L). When analyzed over the complete time course, these were significantly (*P*<0.001, linear regression) higher than those of *mdx-Xist* ^Δhs^ mice with >30% dystrophin (average 1570 U/L). CK levels of mice with 15–30% dystrophin generally fell between those with <15% and >30%, showing a clear correlation between CK and dystrophin levels. CK levels of mice with <15% of dystrophin were similar to *mdx* suggesting that these dystrophin levels were not enough to decrease CK levels (Figure 2B).

In the four limb hanging wire test, *mdx* mice performed significantly (*P*<0.001) worse compared to all the other groups (Figure 2C). *Xist* ^Δhs^ mice were able to hang for the maximum period of time for the entire testing period of 12 weeks, whereas all *mdx-Xist* ^Δhs^ mice performed nearly as well as *Xist* ^Δhs^ mice. Also in the two limb hanging wire test, *Xist* ^Δhs^ and *mdx-Xist* ^Δhs^ mice significantly (*P*<0.001) outperformed *mdx* mice and were able to hang for the maximum allowed time. Performance of *mdx-Xist* ^Δhs^ mice with >30% dystrophin dropped from an age of 9 weeks compared to the other *mdx-Xist* ^Δhs^ mice, but this was due to the poor performance of a single mouse. Generally, the decreased performance was more pronounced in *mdx* mice (Figure 2D). The normalized fore limb grip strength measured for *Xist* ^Δhs^ mice (7.9) was significantly (*P*<0.001) higher than that of *mdx* and *mdx-Xist* ^Δhs^ mice (Figure 2E). Strength was improved towards wild type levels in all *mdx-Xist* ^Δhs^ mice (6.5) and significantly (*P*<0.001) lower in *mdx* mice (5.9). Rotarod performance was significantly (*P*<0.001) better in the *mdx-Xist* ^Δhs^ mice with 15–30% dystrophin compared to all the other groups (Figure 2F). Unfortunately, we observed a high inter group variation, which makes drawing conclusions difficult for this test. Based on these analyses, even the lowest levels of dystrophin analyzed (between 3 and 14%), appear sufficient to improve hanging times and grip strength.

### Dystrophin dependent improvement of histopathology and restoration of gene expression

**Figure 3:**
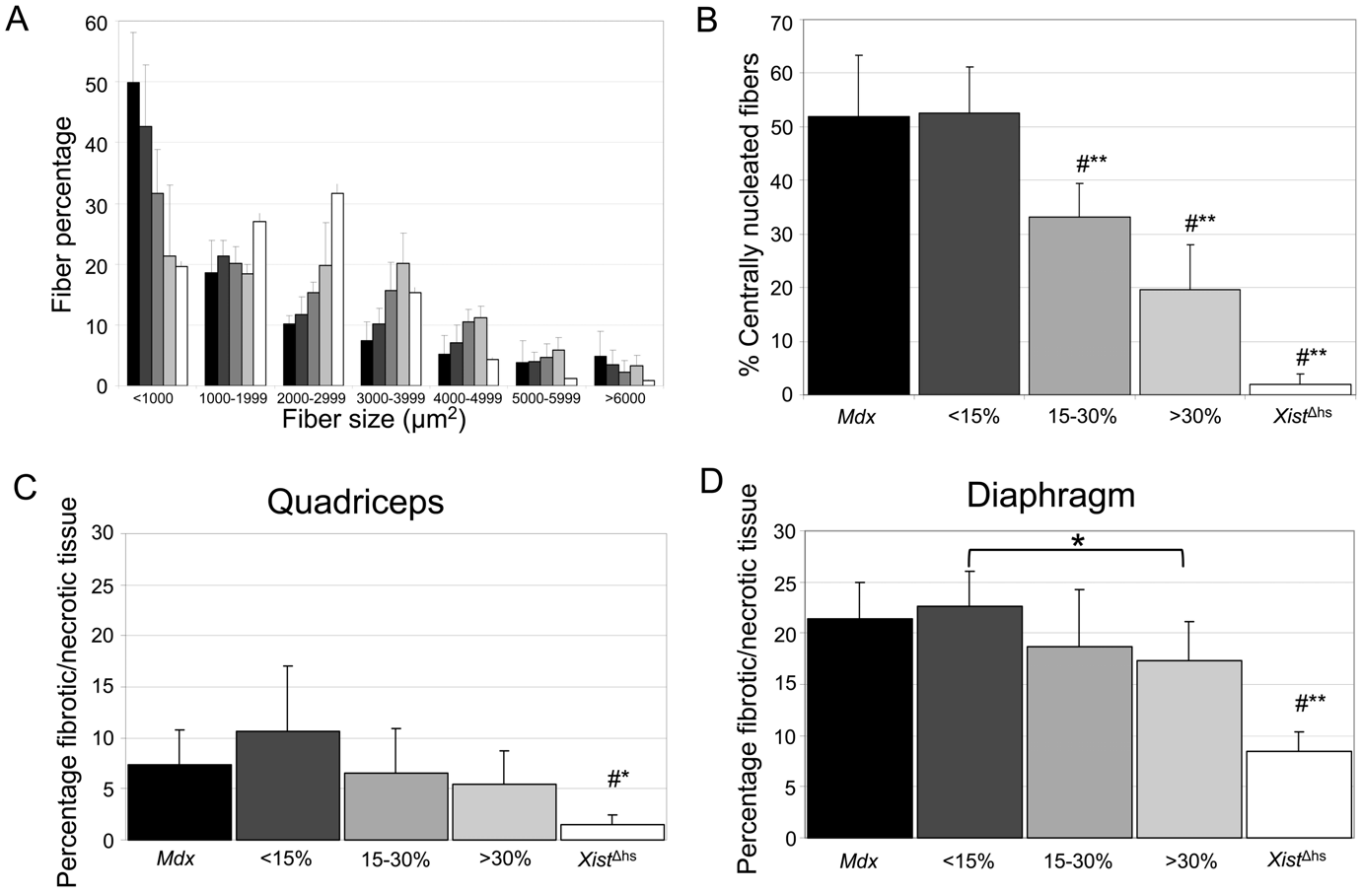
Muscle fiber size, degree of central nucleation and percentage of fibrotic/necrotic tissue. A. Regenerating and hypertrophic fibers were mainly observed in the *mdx* mice. A dystrophin level depend trend towards wild type distribution was observed for *mdx-Xist* ^Δhs^ mice where <15% dystrophin already resulted in improvement. B. Dystrophin levels between 15–30% and >30% resulted in a reduction of the percentage of centralized nuclei of 40% and 60% respectively. C. The quadriceps of all mice was significantly more severely affected compared to *Xist* ^Δhs^ mice. D. The diaphragm was the most severely affected muscle with on average 20% fibrotic/necrotic tissue in *mdx* mice. All mice were significantly more severely affected compared to *Xist* ^Δhs^ mice. *Mdx-Xist* ^Δhs^ mice with >30% dystrophin had less fibrotic/necrotic tissue than *mdx* and *mdx-Xist* ^Δhs^ mice with <15% dystrophin, but this difference was only significant between both *mdx-Xist* ^Δhs^ groups. # indicates a significant difference of that bar with all the other groups. Single asterisks indicate a *P*<0.05 and double asterisks indicate a *P*<0.01.

After sacrificing the animals, the quadriceps, heart and diaphragm were dissected and cryosections were made. To determine fiber size and the percentage of centralized nuclei, the quadriceps of all mice was stained with laminin and DAPI. Five pictures were randomly captured of the middle section of the muscle and analysed with Mayachitra Imago (Figure 3A and B). Regenerating fibers (<1000 µm^2^) and hypertrophic fibers (>6000 µm^2^) were predominantly found in the *mdx* mice (50% and 4.8%, respectively), whereas smaller proportions were observed for the *Xist* ^Δhs^ mice (20% and 0.9%, respectively) and a dystrophin level dependent normalization towards the wild type fiber size distribution was observed for *mdx-Xist* ^Δhs^ mice. The percentage of centralized nucleated fibers was significantly higher (*P*<0.01) in *mdx* (51%) compared to *Xist* ^Δhs^ mice (2%). More than 15% of dystrophin was sufficient to significantly (*P*<0.01) reduce the amount of centralized nuclei by 40%, whereas dystrophin levels above 30% caused a reduction of around 60%.

To determine the percentage of fibrotic/necrotic fibers, sections of the quadriceps, diaphragm and heart were stained with haematoxylin & eosin. For all muscles quantified, except heart, the percentage of fibrosis was significantly higher (*P*<0.001) in *mdx* mice compared to *Xist* ^Δhs^ mice. In the quadriceps of *mdx-Xist* ^Δhs^ mice of the different groups no significant differences was observed, although there appears to be a dystrophin-dependent trend towards lower levels of fibrosis in the mice with moderate levels of dystrophin (Figure 3C). The diaphragm was the most severely affected muscle examined, with fibrotic/necrotic tissue percentages of 20% in both *mdx* and *mdx-Xist* ^Δhs^ mice with <15% dystrophin compared to 8% in *Xist* ^Δhs^ mice. Again, there was a dystrophin-level dependent pattern of reduced fibrotic tissue which reached significance (*P*<0.05) in mice with >30% dystrophin (Figure 3D). Fibrosis/necrosis levels in heart were below 5% in all mice, which is expected, as fibrosis in heart generally is observed in older mice (data not shown) [35].

**Figure 4:**
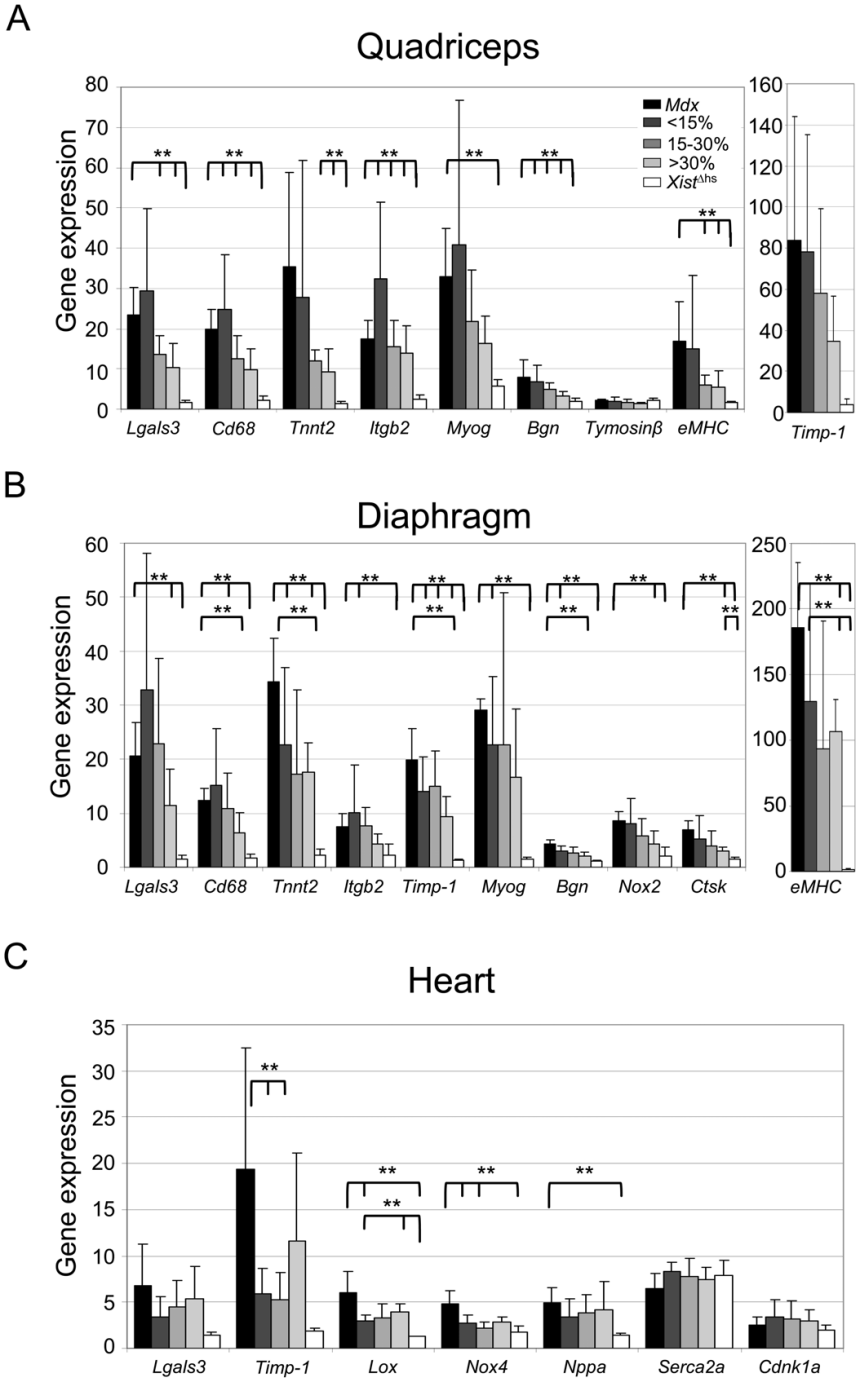
Expression of several genes involved in disease pathology. A and B. For most biomarkers a clear dystrophin level dependent restoration of gene expression levels was observed in the *mdx-Xist* ^Δhs^ mice, where intermediate dystrophin levels resulted in low expression of genes involved in disease pathology. For some genes, dystrophin levels <15% were enough to reduce gene expression while for other genes >30% was necessary. C. In the heart, even dystrophin levels of <15% decreased expression of fibrotic biomarkers like *Timp-1*. Since the mice were young, no difference in expression levels in heart function was observed, except for *Nppa*. Double asterisks indicate a *P*<0.01.

To determine whether low levels of dystrophin were able to normalize the expression of genes known to be involved in inflammation, fibrosis, regeneration and heart function RT-qPCR was performed on mRNA of the quadriceps, diaphragm and heart of all mice [36]. The expression of most genes was significantly (*P*<0.01) elevated in *mdx* mice compared to *Xist* ^Δhs^ mice in the three muscles, and most often normalized in a dystrophin level dependent manner in the *mdx-Xist* ^Δhs^ mice (Figure 4). Notably, in the diaphragm, mRNA levels of the biomarkers for muscle regeneration (*Tnnt2*, *Bgn*) decreased in mice with <15% dystrophin, while biomarkers for inflammation (*Lgals3*, *Cd68*) only decreased in mice with >30% dystrophin. Biomarkers for fibrosis (*Timp-1*, *Nox2*) decreased in mice with <15% and >15% dystrophin, respectively. No difference in expression of heart function genes was observed, except for *Nppa*, which is due to the young age of the mice.

### Dystrophin level dependent protection against exercise induced muscle damage

To determine the protective effect of low dystrophin levels on muscle integrity during chronic exercise, mice were forced to run three times a week on a horizontal treadmill. This was directly followed by a functional test to assess the influence of muscle fatigue on performance. We tested seven *mdx-Xist* ^Δhs^ mice which had an average dystrophin level of 21% (2%–45% median 25.8), six *mdx* and five C57BL/10ScSnJ females (the wild type sub-strain from which the *mdx* mouse was derived).

**Figure 5:**
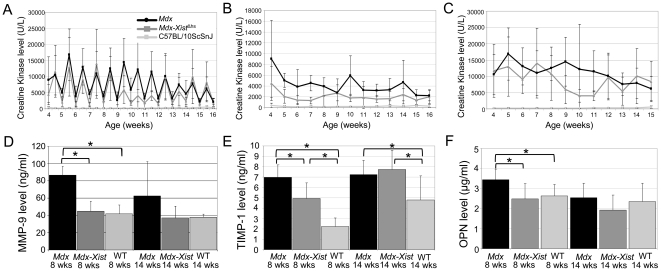
Serum and plasma biomarkers assessed before and directly after treadmill running. A. CK levels assessed before and directly after treadmill running. CK levels collected before exercise were significantly (*P*<0.01) lower than those collected directly after exercise in *mdx-Xist* ^Δhs^ and *mdx* mice. This exercise induced increase was absent in wild type mice. B. CK levels determined before exercise were significantly (*P*<0.01) elevated in *mdx* and *mdx-Xist* ^Δhs^ mice compared to wild type, but this was less pronounced for the *mdx-Xist* ^Δhs^ mice as *mdx* mice had significantly (*P*<0.05) higher CK levels. C. Plasma collected directly after exercise contained extremely high CK levels, in both *mdx* and *mdx-Xist* ^Δhs^ mice but not in wild type mice. D. Serum levels of MMP-9 were elevated in *mdx* mice compared to *Xist* ^Δhs^ mice at both 8 and 14 weeks of age, while levels were normalized in *mdx-Xist* ^Δhs^ mice. E. TIMP-1 levels were elevated in serum of both *mdx* and *mdx-Xist* ^Δhs^ mice. In wild type mice we found an age related increase of the serum TIMP-1 level. Interestingly, at the age of 8 weeks, levels of *mdx-Xist* ^Δhs^ mice were significantly lower than those of *mdx* mice. F. *Mdx* mice had significantly elevated OPN levels compared to both wild type and *mdx-Xist* ^Δhs^ at 8 weeks of age. Single asterisks indicate a *P*<0.05, average dystrophin level of *mdx-Xist* ^Δhs^ mice was 21% (2%–45% median 25.8).

CK levels were assessed on Mondays before exercise (Figure 5B) and on Fridays directly after exercise (Figure 5C). For *mdx* and *mdx-Xist* ^Δhs^ mice CK levels increased significantly (*P*<0.01) upon exercise (4150 U/L before versus 11170 U/L after for *mdx* and 2070 U/L before versus 8850 U/L after for *mdx-Xist* ^Δhs^), whereas this was not observed for the wild type mice (250 U/L before versus 291 U/L after) (Figure 5A). CK levels of the *mdx-Xist* ^Δhs^ mice were significantly (*P*<0.05) lower compared to *mdx*, both before and after exercise. Wild type mice had significantly (*P*<0.01) lower CK levels than those of *mdx* and *mdx-Xist* ^Δhs^ mice. *Mdx-Xist* ^Δhs^ mice gained significantly (*P*<0.01) less weight over time than *mdx* mice, as they had a higher body weight at the start, but a similar body weight at the end of the test regime (data not shown). Wild type mice were significantly (*P*<0.01) less heavy than *mdx* mice, but did not significantly differ in weight from the *mdx-Xist* ^Δhs^ mice.

At an age of 8 and 14 weeks, serum levels of matrix metalloproteinase-9 (MMP-9), tissue inhibitors of matrix metalloproteinase-1 (TIMP-1) and osteopontin (OPN) were assessed. These markers have been identified as potential biomarkers to monitor disease progression in DMD patients [37]. For MMP-9, 8 week old *mdx* mice had significantly (*P*<0.05) higher levels when compared to wild type and *mdx-Xist* ^Δhs^ mice (Figure 5D). No significant difference was observed between *mdx-Xist* ^Δhs^ and wild type mice. TIMP-1 levels were significantly (*P*<0.05) elevated in both *mdx* and *mdx-Xist* ^Δhs^ mice compared to wild type mice at the age of 8 and 14 weeks (Figure 5E). Interestingly, a significant (*P*<0.05) difference was also observed between *mdx* and *mdx-Xist* ^Δhs^ mice aged 8 weeks. OPN levels were significantly (*P*<0.05) elevated in *mdx* compared to both wild type and *mdx-Xist* ^Δhs^ at 8 weeks. This difference was not observed at 14 weeks of age (Figure 5F).

**Figure 6:**
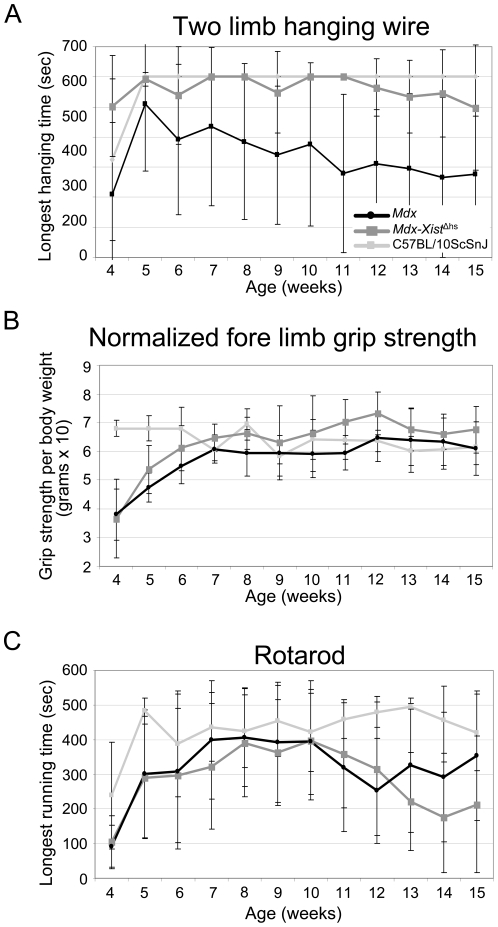
Functional performance measured directly after treadmill exercise. A. Longest hanging time with the two limb hanging test was achieved by wild type and *mdx-Xist* ^Δhs^ mice which both performed significantly (*P*<0.001) better than *mdx* mice. B. Fore limb grip strength of *mdx-Xist* ^Δhs^ mice was significantly (*P*<0.001) better than that of *mdx* mice. C. Wild type mice outperformed *mdx* and *mdx-Xist* ^Δhs^ mice on the rotarod, while no significant difference was observed between *mdx* and *mdx-Xist* ^Δhs^ mice. Average dystrophin level of *mdx-Xist* ^Δhs^ mice was 21% (2%–45% median 25.8).

The chronic treadmill exercise protocol was completed by all mice without any problems. However, we later found that fibrosis was much worse in these exercised mice and that the low dystrophin levels were not enough to prevent worsening of the muscle's condition (see below). Directly after treadmill exercise, mice were subjected to two limb hanging wire test, fore limb grip strength or rotarod (Figure 6). For the two limb hanging wire test, the hanging time of *mdx* mice decreased over time, and was significantly (*P*<0.001) lower than those of the *mdx-Xist* ^Δhs^ and wild type mice. The hanging time of the *mdx-Xist* ^Δhs^ and wild type mice did not differ significantly. Performance of the grip strength was consistently lower for the *mdx* mice (5.8) than for the other groups (*mdx-Xist* ^Δhs^ 6.3 and wild type 6.4). Wild type mice performed well at the start of the functional test regime, but their strength slightly decreased over time. Grip strength of *mdx* and *mdx-Xist* ^Δhs^ mice followed the same curve, and *mdx-Xist* ^Δhs^ mice performed significantly (*P*<0.001) better than *mdx* mice. Rotarod running time was significantly (*P*<0.001) higher in the wild type mice with an average running time of 430 seconds, which was generally 130 seconds longer than that of both dystrophic mouse strains. The performance of *mdx* and *mdx-Xist* ^Δhs^ mice did not significantly differ for this test.

### Low dystrophin levels did not fully prevent exercise induced pathology

**Figure 7:**
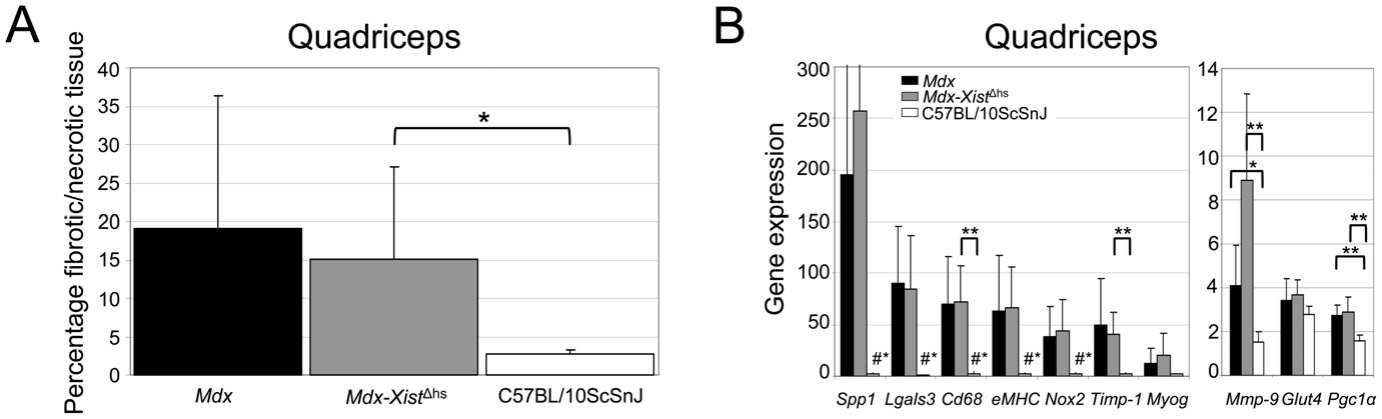
Exercise induced histopathology and biomarker gene expression. A. Wild type mice were less severely affected than *mdx* and *mdx-Xist* ^Δhs^ mice. Due to high variation between individual *mdx* mice, the difference between *mdx* and wild type mice was not significant. *Mdx-Xist* ^Δhs^ mice had slightly less fibrotic tissue than *mdx* mice. B. No difference in expression of genes involved in disease pathology was found between *mdx* and *mdx-Xist* ^Δhs^ mice. Both mouse strains did differ significantly from wild type mice, of which the expression levels were low. Single and double asterisks indicate a *P*<0.05 and *P*<0.01, respectively. # Indicates a significant difference from all other groups, average dystrophin levels of *mdx-Xist* ^Δhs^ mice was 21% (2%–45% median 25.8).

The effect of the chronic exercise regime on histopathology was assessed by measuring the percentage of fibrotic/necrotic tissue for the quadriceps. On average, 18% of the quadriceps of the *mdx* mice consisted of fibrotic/necrotic tissue, compared to less than 5% for wild type mice. *Mdx-Xist* ^Δhs^ mice had slightly more healthy tissue than *mdx* mice (Figure 7A). When compared to fibrosis observed in mice which underwent the functional test regime without treadmill running, histopathology was much worse. At the end of the chronic exercise regime, Evans blue dye was injected intravenously and the percentage of Evans blue dye positive fibers was manually counted for the quadriceps of all mice. Hardly any Evans blue dye positive fibers were detected in wild type muscle (0.41%), while both in *mdx* and *mdx-Xist* ^Δhs^ mice 11.11% and 9.23% of positive fibers were found. This suggests that while low dystrophin levels are sufficient to improve muscle function and muscle fiber integrity in the absence of chronic exercise, they apparently are not sufficient to protect against fiber damage induced by the chronic treadmill exercise regime.

The expression of some fibrosis, inflammation and regeneration biomarkers was assessed for the quadriceps. For most genes, *mdx* and *mdx-Xist* ^Δhs^ mice had significantly (*P*<0.05) increased expression when compared to wild type levels. However, between *mdx* and *mdx-Xist* ^Δhs^ mice no difference in expression was observed indicating that the dystrophin levels in these mice were not sufficient to prevent pathology induced by chronic exercise (Figure 7B). In addition, we were able to confirm the differences observed in serum for MMP-9, TIMP-1 and OPN (*Spp1*) on gene expression level.

## Discussion

With several potential therapeutic compounds being tested in clinical settings for DMD, there is an increasing need to gain detailed knowledge about which levels of dystrophin are of therapeutic value and which and/or how low dystrophin levels affect the different pathological pathways. This would help the optimization phase of pre-clinical research on therapeutic approaches aiming at dystrophin restoration. It will also facilitate providing patients and parents who are candidates to participate in clinical trials for these approaches with realistic expectations.

Several attempts to generate mice expressing low dystrophin levels have been undertaken previously, unfortunately without success since none of these strains expressed a wide variety of low dystrophin levels in the most interesting range. With no accessibility to a good model, current knowledge and rough estimations are based on human female DMD carriers, heterozygous *mdx* mice, generated transgenic mice expressing lower levels of full-length or truncated dystrophin of murine or human origin and antisense oligonucleotide (AON) treated *mdx* mice.

In this paper, we describe the easy to generate *mdx-Xist* ^Δhs^ mouse model, which facilitates assessment of the effects of different dystrophin levels on muscle performance and integrity. Mice with very low (<15%), low (15–30%) and moderate dystrophin (>30%) levels showed a dystrophin level dependent reduction of the homolog utrophin. All *mdx-Xist* ^Δhs^ mice had a normalized body weight and outperformed *mdx* mice in both hanging wire tests and in the grip strength test, indicating that even low dystrophin levels (<15%) can bring about improved performance in this mouse model. The improved functional performance observed is in concordance with that previously described in *mdx^3cv^* mice and AON treated mice expressing low levels of dystrophin [29], [38]. Although, ∼5% of dystrophin in the *mdx^3cv^* mice resulted in increased specific tetanic force and partly resistance to eccentric contractions in young, but not in old mice, histopathology was not different from dystrophin negative *mdx^4cv^* mice. This again is in line with our observation in *mdx-Xist* ^Δhs^ mice expressing <15% dystrophin, were we also found no histopathological improvements, while we did find improvements on motor function. Dystrophin levels >30% reduced CK levels significantly while for lower percentages only a non-significant trend was found. Also for the expression of pro-inflammatory biomarkers and histopathology, a dystrophin level dependent improvement was observed where for some genes >15% of dystrophin led to improvement while for other genes >30% dystrophin was needed. In chronically exercised mice with ∼20% dystrophin, normalization towards wild type levels was observed for three different disease serum markers (CK, MMP-9 and TIMP-1). This confirms findings that MMP-9 and TIMP-1 are useful serum biomarkers to monitor disease pathology in DMD patients' serum [37]. Even after treadmill exercise the *mdx-Xist* ^Δhs^ mice performed significantly better than the *mdx* mice for the two limb hanging wire test and grip strength. However, the levels of fibrosis and Evans blue dye uptake were similar for *mdx-Xist* ^Δhs^ mice and *mdx* mice, indicating that ∼20% of dystrophin is not sufficient to protect against forced exercise induced damage. Thus, while even dystrophin levels below 15% can improve pathology and performance, levels of >20% are needed to fully protect muscle fibers from exercise-induced damage.

The fact that most of our observations concur with those of other studies conducted in transgenic mice and AON treated mice confirms the usefulness of the *mdx-Xist* ^Δhs^ mouse as a dystrophic mouse strain. It should be noted that the *mdx-Xist* ^Δhs^ mouse expresses dystrophin in a random manner where nuclei either express wild type levels of dystrophin or no dystrophin at all. This is similar to the pattern what is expected for cell therapy, gene therapy and to what is observed in transverse cross sections after phosphorodiamidate morpholino oligomer (PMO) administration [12], but different from that of 2′-O-methyl phosphorothioate (2OMePS) [11] or gentamicin administration [39], which result in a more evenly low dystrophin expression in nearly all fibers. With detailed knowledge lacking regarding the migration properties of AONs and dystrophin over the membrane, it is unknown whether the mosaic pattern seen in longitudinal cross sections of *mdx-Xist* ^Δhs^ mice could be expected upon PMO administration. The effect of differences in distribution of dystrophin has never been studied in great detail; i.e. whether 20% of dystrophin in all muscles is better than 100% dystrophin in 20% of muscle fibers. Previously, Phelps et al. showed that mice expressing dystrophin 10-fold greater than wild type levels in a mosaic manner had 34% of centralized nuclei, while mice expressing similar amounts uniformly only had 7% of central nuclei. More detailed studies especially in mice expressing low dystrophin levels are needed and could easily be conducted using our mouse model and comparing them to 2OMePS or gentamicin treated *mdx* mice [25]. Using the *mdx-Xist* ^Δhs^ mouse as study object is favored above treated *mdx* mice since no expensive treatment is needed to achieve a range of low dystrophin levels. Notably, we observed variation in pathology for mice with similar levels of dystrophin. This phenomenon is also anticipated for patients currently in clinical trials aiming at dystrophin restoration. Our mouse model will allow extended studies to elucidate this discrepancy.

In summary, the Gaussian distribution of low dystrophin levels obtained in the *mdx-Xist* ^Δhs^ mouse and the ease of generating large numbers of mice, make it a good mouse model for more detailed research on the effect of low levels of dystrophin on several aspects of DMD pathology and effects of future treatment strategies for dystrophinopathies.

## Materials and Methods

### Ethics statement

All experiments were approved by and performed following the guidelines of the Dier Experimenten Commissie (Animal Experimental Commission) of the Leiden University Medical Center (Permit Numbers: 08096 and 09136). Effort was put in minimizing the amount of distress caused to the animals as much as possible.

### Animal care

All studied mouse strains were bred at the animal facility of the LUMC where they were housed in individually ventilated cages with 12-h light-dark cycles. The *Xist* ^Δhs^ model [32] was a kind gift from Prof Brockdorff (MRC Clinical Sciences Centre London, UK, current affiliation Department of Biochemistry, University of Oxford, UK). Mice were given standard chow and water *ad libitum* and were weighed two times a week. Breeding pairs of *mdx* (C57BL/10ScSn-mdx/J) males and *Xist* ^Δhs^ females gave birth to *mdx-Xist* ^Δhs^ females (Figure 1A). Genotyping was performed on DNA obtained from tail tips by PCR analysis to confirm the mutation in the *Dmd* and *Xist* gene (primers and PCR conditions on request).

### Functional test regimes

To test the functional abilities of the different mouse strains over time, a group of 5 *mdx*, 24 *mdx-Xist* ^Δhs^ (0–15% dys *n* = 9, 15–30% dys *n* = 9 and >30% *n* = 6, as assessed by Western blot of quadriceps muscles at the end of the study) and 5 *Xist* ^Δhs^ females underwent a functional test regime consisting of four different functional tests starting at four weeks of age, as described previously [40]. Plasma creatine kinase (CK) levels were determined once a week at the beginning of the functional test regime. Mice were sacrificed by cervical dislocation after 12 weeks of testing and muscles were isolated.

To determine whether functional test performances could be influenced by chronic treadmill exercise, 6 *mdx*, 7 *mdx-Xist* ^Δhs^ and 5 C57BL/10ScSnJ four week old females had to run 30 minutes on a horizontal treadmill at 12 m/min, directly followed by either fore limb grip strength, rotarod running or the two limb hanging wire test three times a week for 12 weeks. CK levels were determined two times a week; on Mondays before exercise and on Fridays directly after the two limb hanging wire test. At the age of 8 and 14 weeks additional blood was collected via the tail to assess several serum biomarkers (MMP-9, TIMP-1 and OPN) of which we have determined in parallel research that these correlate with disease severity [37]. After the 12 week chronic exercise protocol, mice were forced to run a final time on the horizontal treadmill for 30 minutes at 12 m/min. Evans blue dye (5 µg/µl) was applied intravenously (5 µl/gram of body weight) 20–30 minutes after this exercise. Twenty four hours after the injection, mice were sacrificed by cervical dislocation and muscles were isolated. Standardized operating procedures from the TREAT-NMD network were implemented for grip strength, both hanging wire tests and treadmill running (http://www.treat-nmd.eu/resources/research-resources/dmd-sops/).

### Fore limb grip strength test

Fore limb grip strength was assessed by means of a grip strength meter (Columbus Instruments, USA). Mice were tested five times, with three consecutive measurements per trial (15 in total), and a two minute interval between trials. The three highest measured values were averaged to calculate the absolute strength, which was divided by the body weight in grams.

### Rotarod

Mice were placed on the Rotarod (Ugo Basile, Italy) that accelerated from 5 to 45 rotations per minute within 15 seconds. The test session ended when a mouse ran for 500 seconds without falling. Mice that fell off before 500 seconds were given a maximum of two more tries. The longest running time was used for analysis.

### Two limb hanging wire test

Mice were suspended above a metal cloth hanger secured above a cage and released a few seconds after instinctively grasping the wire with the fore limbs. Depending on the functional ability of the mouse, 2–4 limbs and the tail were used during a 10 minute hanging session. Mice that fell down before the 10 minute time limit were given two more tries. The longest hanging time was used for further analysis.

### Four limb hanging wire test

The start position of this test was with all limbs. To this end, mice were placed on a grid, which was then turned upside down above a cage filled with bedding. The session ended after a hanging time of 10 minutes was achieved or otherwise after three sessions. The longest hanging time was used for further analysis.

### Blood CK, MMP-9, TIMP-1 and OPN level analysis

For CK determination, blood was collected via a small cut at the end of the tail in a Minicollect tube (0.8 ml Lithium Heparin Sep, Greiner bio-one, Austria). Plasma CK levels were determined with Reflotron CK test strips in the Reflotron plus machine (Roche diagnostics Ltd, UK) at the day of collection.

To detect MMP-9, TIMP-1 and OPN levels, blood was collected via the tail and allowed to clot at room temperature for 10 minutes before spinning (1700 *g* for 10 minutes at 4°C). The serum supernatant was carefully aspirated and stored at −80°C, before use. To determine serum biomarker levels in mouse, the mouse MMP-9 (total) immunoassay kit (catalog number MMPT90) and mouse TIMP-1 immunoassay kit (catalog number MTM100) were purchased from R&D systems (Abingdon, United Kingdom), while the mouse OPN kit (code number 27351) was purchased from IBL (Hamburg, Germany). Experiments were performed as per manufacturer's protocol.

### Histological examinations

For all 16 week-old mice the quadriceps, gastrocnemius, tibialis anterior, triceps, biceps, diaphragm and heart were dissected and snap frozen in 2-methylbutane (Sigma Aldrich, the Netherlands) cooled in liquid nitrogen. Cross-sections of 8 µm were cut on Superfrost Plus slides (Thermo Fishes Scientific, Menzel-Gläser, Germany) with a Shandon cryotome (Thermo Fisher Scientific Co., USA) along the entire length of the muscle with an interval of 240 µm between the sections. The excess tissue between the sections was collected in MagNa Lyser Green Beads tubes (Roche diagnostics Ltd, UK) for total RNA and protein isolation.

To determine the percentage of fibrotic/necrotic fibers, sections of the quadriceps were stained with Harris Haematoxylin and Eosin (H&E) (Sigma Aldrich, the Netherlands) and examined with a light microscope (Leica DM LB, Leica Microsystems, the Netherlands) at a 5 times magnification and images were captured with a Leica DC500 camera and Leica IM50 software (Leica Microsystems, the Netherlands) from the entire middle section of the muscle. Analysis was performed in a double-blinded manner, by two independent researchers on stitched pictures with the color deconvolution plugin of the ImageJ software (Rasband, W.S., ImageJ, U. S. National Institutes of Health, Bethesda, Maryland, USA, http://rsb.info.nih.gov/ij/, 1997–2008) as described previously [40]. Quadriceps, tibialis anterior, diaphragm and heart sections were stained with dystrophin (C-20, dilution 1∶50, Santa Cruz, Germany) and donkey-anti-goat Alexa 488 (dilution 1∶1000, Invitrogen, the Netherlands) and mounted with Vectashield mounting medium including DAPI (Vector Labs, Germany). The percentage of dystrophin positive fibers was manually and independently counted by two persons in a blinded manner for the quadriceps, stained with dystrophin and laminin (ab11575, dilution 1∶50 Abcam, UK) where donkey-anti-goat Alexa 488 and donkey-anti-rabbit Alexa 594 (dilution 1∶1000, Invitrogen, the Netherlands) were respectively used as a secondary antibody. To determine the dystrophin expression per nucleus, longitudinal sections of the quadriceps were cut and stained with dystrophin, spectrin (dilution 1∶200, Thermo Scientific, USA) and DAPI. Co-localization of dystrophin with nNOS (H-299, dilution 1∶50, Santa Cruz, Germany) and β-dystroglycan (NCL β-DG, dilution 1∶50, Novocastra, UK) was determined in the quadriceps. The donkey-anti-rabbit Alexa 488 (dilution 1∶1000, Invitrogen, the Netherlands) and MOM kit (Vector Laboratories, UK) were used as secondary antibody respectively.

Fiber size and the percentage of centrally nucleated fibers were determined with Mayachitra Imago 1 (http://www.mayachitra.com/imago/) on five randomly captured images of the quadriceps stained with laminin and DAPI (van Putten et al. manuscript in preparation). Fibers were segmented based on intensity differences between the membrane and the cytoplasm. The cross sectional area of each segmented fiber was computed by the software. Centrally nucleated fibers were identified based on the maximum intensity of the cytoplasm which is higher in fibers with a centralized nucleus than without. The percentage of Evans blue dye positive fibers was manually counted in a blinded manner for the quadriceps of all mice which were subjected to the chronic treadmill exercise. All fluorescent stained sections were examined with a fluorescent microscope (Leica DM RA2) at a 16 times magnification and six images were randomly captured with a Leica DC350FX snapshot camera.

### Western blot analysis

Muscles were homogenized in treatment buffer (100 mM Tris-HCl pH 6.8 and 25% SDS) using MagNa Lyser green beads tubes in the MagNa Lyser (Roche Diagnostics, the Netherlands). Protein concentration was determined using the BCA protein assay kit (Thermoscientific, USA), according to the manufacturer's instructions. Subsequently, the homogenate was complemented to contain 75 mM Tris-HCl pH 6.8, 15% SDS, 5% β-mercaptoethanol, 20% glycerol and 0.001% bromophenol blue. Samples were boiled for 5 minutes and 50 µg was loaded on a hand-made 4–7% gradient polyacrylamide gel and run overnight at 4°C. The gel was blotted to nitrocellulose BA83 (Whatman, Schleicher & Schuell, Germany) for 6 hours at 4°C. The blot was blocked with 5% non-fat dried milk (Campina Melkunie, the Netherlands) in Tris Buffered Saline (TBS) plus 0.05% Tween20 followed by an overnight incubation with NCL-DYS1 (dilution 1∶125, Novacastra, UK) or MANCHO3 (dilution 1∶50,Tebu-bio, the Netherlands) in TBS plus 0.05% Tween20 to detect dystrophin and utrophin respectively. Myosin (MF20 dilution 1∶20000, Developmental Studies Hybridoma Bank, University of Iowa, Iowa City) was used as a loading control. The fluorescent IRDye 800CW goat anti-mouse IgG (dilution 1∶5000 for dystrophin and utrophin, dilution 1∶10000 for myosin, Li-Cor, USA) was used as a secondary antibody. Blots were visualized and quantified with the Odyssey system and software (Li-Cor, USA) as described previously [41]. For each sample, 2–9 technical replicates were performed. A standard deviation of 15% was allowed between the replicates.

### RT-qPCR analysis

Total RNA was isolated with RNA-Bee (Tel-Test, Bio-Connect, the Netherlands) and purified with the NucleoSpin RNA II kit including a DNAse digestion (Bioke, the Netherlands) according to the manufacturer's instructions. The RNA concentration was measured on a Nanodrop (Nanodrop Technologies, USA) and integrity was checked with a total RNA nano chip assay on a labchip assay (Agilent, the Netherlands). cDNA was synthesised with random hexamer primers and gene expression levels were determined by Sybr Green based Real Time qPCR (95°C 10 sec, 60°C 30 sec, 72°C 20 sec. 45 cycles followed by melting curve determination) on the Roche Lightcycler 480 (Roche diagnostics Ltd, UK). Expression of genes involved in inflammation (*Lgals3, Cd68*), fibrosis (*Tnnt2, Itgb2, Timp-1, Nox2, Lox, Nox4, Mmp9, Spp1*) regeneration (*Myog, Bgn, eMHC, Ctsk, Thymosinβ*), heart function (*Nppa, Serca2a, Cdnk1a*) and exercise (*Glut4, Pgc1α*) were analysed. *Gapdh* was used as a reference gene, since the expression of this gene did not differ between different muscles or over time. Primer efficiencies were determined with LinREgPCR version 11.1 [42]. The C_p_ values were obtained with the second derivative maximum method and analysed.

### Statistics

Statistical analyses were performed with statistical software in R (version R2.11.1). *Mdx-Xist* ^Δhs^ mice were divided in three groups by k-means clustering, based on the median level of dystrophin quantified by Western blot data (*n* = 2–9). Figures 2, 5 and 6 summarize the data, i.e. showing the mean and standard deviation per genotype and age. To overcome applying separate tests for each age between genotypes, which suffers from multiple testing and ignores the age trend, we applied an analysis of covariance (ANCOVA) to the data. ANCOVA was applied to temporal functional performance, body weight and CK level data with age as continuous and genotype as a categorical variable. In choosing the appropriate model we applied the principle of parsimony. Given the application of tests for several different variables, we considered *P*<0.01 as significant.

The two-tailed homoscedastic Student's t-test was conducted for comparison of the single time point histological, serum biomarker and gene expression data. *P*<0.05 was considered significant for all tests, while *P*<0.01 was used for the gene expression data to correct for multiple testing.

## Supporting Information

Figure S1 **Dystrophin expression in** ***mdx-Xist*** **^Δhs^ mice.** A. Example of a Western blot of some skeletal muscles and heart. In order to determine the expression levels of the different muscles, a concentration curve was made of wild type samples from the corresponding muscle. Myosin was used as a loading control. B. Relative dystrophin levels of skeletal muscles compared to these of the quadriceps. Dystrophin levels of six *mdx-Xist* ^Δhs^ mice, with dystrophin levels of <15%, 15–30% and >30%, were assessed for skeletal muscles and heart by Western blot. Levels are expressed relative to the quadriceps. Those of gastrocnemius and triceps were similar to quadriceps levels, while those of the tibialis anterior and the biceps were slightly higher. Low levels were observed for the diaphragm and heart. qua: quadriceps, gas: gastrocnemius, ta: tibialis anterior, tri: triceps, bi: biceps, dia: diaphragm, ha: heart. C. Small groups of dystrophin positive fibers were randomly distributed over the muscles. Representative pictures of *mdx-Xist* ^Δhs^ mice with 8 and 23% dystrophin.
(TIF)

Figure S2 **Dystrophin co-localisation with β-dystroglycan and nNOS in** ***mdx-Xist*** **^Δhs^ mice.** A. Dystrophin co-localises with β-dystroglycan (top) and nNOS (bottom). B. For *mdx-Xist* ^Δhs^ mice dystrophin levels previously determined on several Western blots (representative blot shown in Figure 1B) were compared to utrophin levels. The relative level of utrophin nicely correlated to that of dystrophin. Mice with intermediate dystrophin levels had decreased utrophin levels (lane 2 and 6) while mice with low dystrophin levels had higher utrophin levels (lane 1, 3–5). *Mdx/utrn* mice expressing utrophin on zero, one or two alleles were taken along as controls. Myosin was used as a loading control.
(TIF)
