# Supplementary figures and images for: The Effects of Low Levels of Dystrophin on Mouse Muscle Function and Pathology
Source: PLoS One. 2012 Feb 16;7(2):e31937. doi: 10.1371/journal.pone.0031937 (PMC3281102; doi:10.1371/journal.pone.0031937)

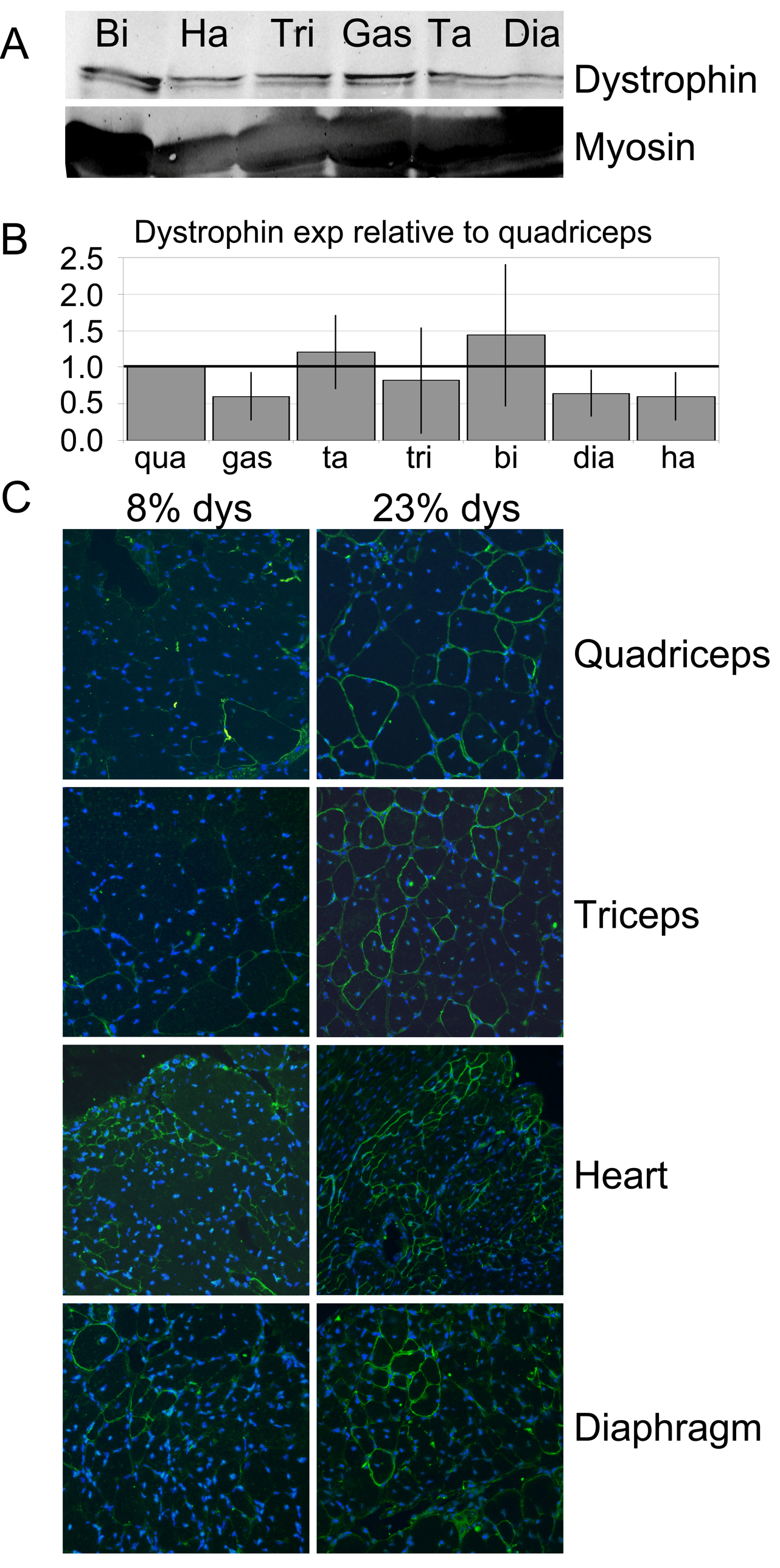

Supplement: Figure S1 — Dystrophin expression in mdx-Xist Δhs mice. A. Example of a Western blot of some skeletal muscles and heart. In order to determine the expression levels of the different muscles, a concentration curve was made of wild type samples from the corresponding muscle. Myosin was used as a loading control. B. Relative dystrophin levels of skeletal muscles compared to these of the quadriceps. Dystrophin levels of six mdx-Xist Δhs mice, with dystrophin levels of <15%, 15–30% and >30%, were assessed for skeletal muscles and heart by Western blot. Levels are expressed relative to the quadriceps. Those of gastrocnemius and triceps were similar to quadriceps levels, while those of the tibialis anterior and the biceps were slightly higher. Low levels were observed for the diaphragm and heart. qua: quadriceps, gas: gastrocnemius, ta: tibialis anterior, tri: triceps, bi: biceps, dia: diaphragm, ha: heart. C. Small groups of dystrophin positive fibers were randomly distributed over the muscles. Representative pictures of mdx-Xist Δhs mice with 8 and 23% dystrophin. (TIF) [file pone.0031937.s001.tif]

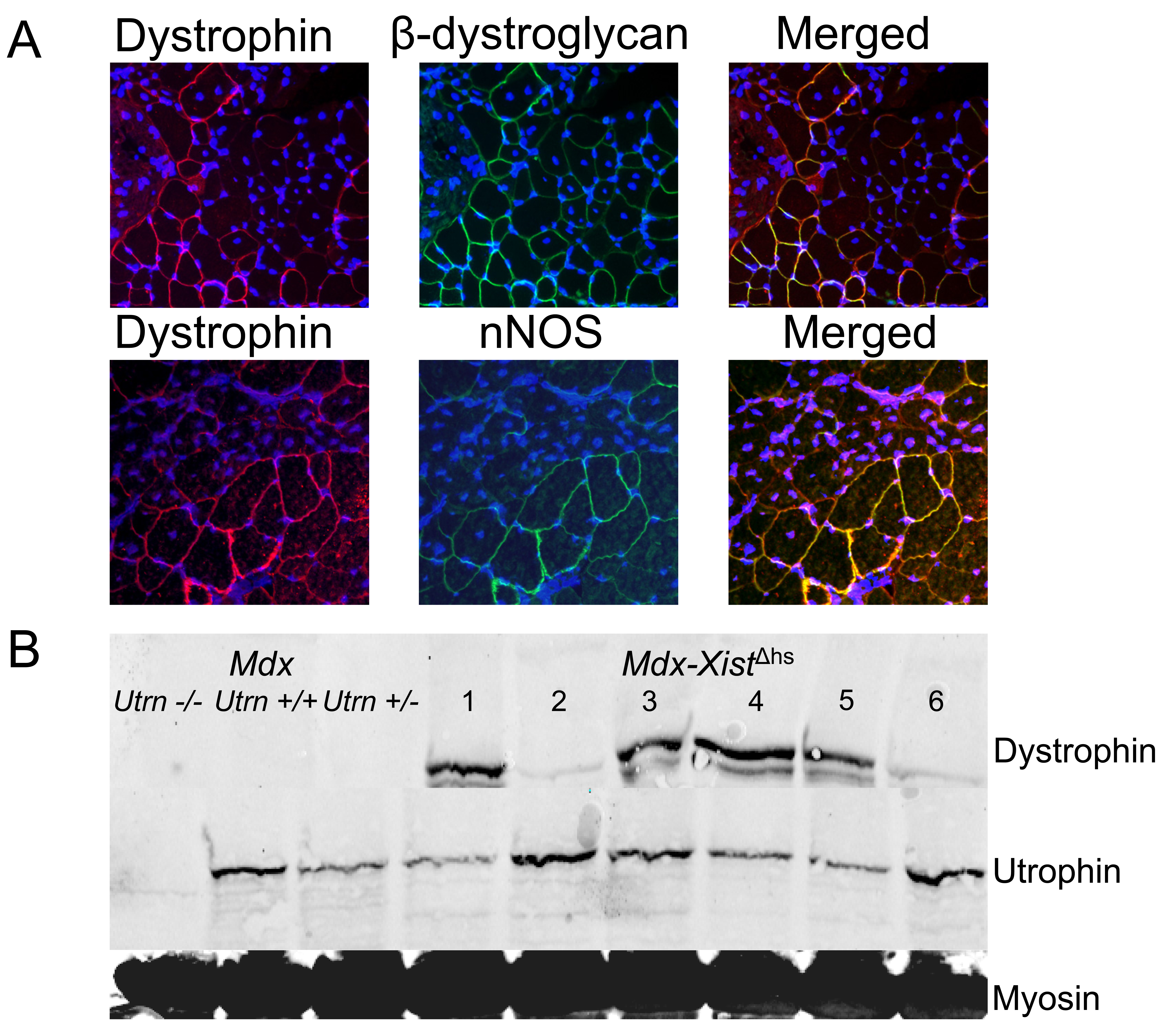

Supplement: Figure S2 — Dystrophin co-localisation with β-dystroglycan and nNOS in mdx-Xist Δhs mice. A. Dystrophin co-localises with β-dystroglycan (top) and nNOS (bottom). B. For mdx-Xist Δhs mice dystrophin levels previously determined on several Western blots (representative blot shown in Figure 1B) were compared to utrophin levels. The relative level of utrophin nicely correlated to that of dystrophin. Mice with intermediate dystrophin levels had decreased utrophin levels (lane 2 and 6) while mice with low dystrophin levels had higher utrophin levels (lane 1, 3–5). Mdx/utrn mice expressing utrophin on zero, one or two alleles were taken along as controls. Myosin was used as a loading control. (TIF) [file pone.0031937.s002.tif]
